# Role of Cardiac Magnetic Resonance (CMR) in Determining Implantable Cardioverter-Defibrillator (ICD) Indication in Acromegalic Cardiomyopathy: A Case Report

**DOI:** 10.7759/cureus.32234

**Published:** 2022-12-05

**Authors:** Naoto Inoue, Hiroya Hayashi, Shoichi Ehara, Yasuhiro Izumiya, Daiju Fukuda

**Affiliations:** 1 Cardiovascular Medicine, Osaka Metropolitan University Graduate School of Medicine, Osaka, JPN

**Keywords:** coronary artery disease, fatal arrhythmia, cardiac magnetic resonance, implantable cardioverter-defibrillator, acromegalic cardiomyopathy

## Abstract

Potentially fatal arrhythmia is one of the causes of premature death in acromegalic cardiomyopathy patients who are not properly treated. Implantable cardioverter-defibrillator (ICD) is one of the most effective and established treatments to prevent sudden cardiac death due to potentially fatal tachyarrhythmia. However, since the indication of ICD changes depending on whether it is ischemic cardiomyopathy or non-ischemic cardiomyopathy, proper diagnosis is important in patients with these diseases. Cardiac magnetic resonance (CMR) is a convenient and useful tool for diagnosing these. Both potentially fatal arrhythmias and coronary artery disease (CAD) are known to be important complications of acromegaly. Herein, we present a case of potentially fatal tachyarrhythmia due to acromegalic cardiomyopathy with the acute coronary syndrome. Furthermore, we mention the usefulness of CMR in the case which is difficult to determine the indication for an ICD.

## Introduction

Cardiovascular disease is a principal cause of premature mortality in acromegalic patients. The fundamental physiologic derangements in acromegaly are a pathologically elevated serum concentration of growth hormone (GH), and insulin-like growth factor-1 (IGF‐1) [1]. Long-term elevations in serum GH and IGF-I levels can cause myocardial hypertrophy and interstitial fibrosis of cardiomyocytes [2]. The high prevalence of arrhythmias and sudden cardiac death in acromegalic patients may be related to myocardial interstitial fibrosis [3]. An implantable cardioverter-defibrillator (ICD) should be implanted for secondary prevention, as the source of potentially fatal arrhythmias often seen in these patients is myocardial fibrosis and scarring, as a result of the underlying acromegalic pathology [1]. It is important to accurately diagnose the cause of potentially fatal arrhythmias, and for such a purpose, cardiac magnetic resonance (CMR) is useful as it evaluates cardiac mass, function, and fibrosis. Herein, we present a case of ventricular fibrillation (VF) due to acromegalic cardiomyopathy but with coexisting coronary artery disease (CAD).

## Case presentation

 We report the case of a 69-year-old Japanese male, who suffered out-of-hospital cardiac arrest (OHCA) due to ventricular fibrillation (VF) caused by acromegalic cardiomyopathy, despite undergoing appropriate treatment. His medical history included a surgical resection of a pituitary adenoma for acromegaly at the age of 60 years, and subsequent octreotide long-acting repeatable therapy, after which his serum insulin-like growth factor-1 (IGF-1) levels were under control. He was noted to have diminished left ventricular (LV) function; however, he had no symptoms of dyspnea. After ruling out other secondary cardiomyopathies, he was diagnosed with LV dysfunction due to acromegaly cardiomyopathy. A transthoracic echocardiogram (TTE) showed a gradual improvement of his ejection fraction (EF) value from 34% to 43% over a period of nine years (Table 1), after receiving optimized heart failure medication such as beta-blockers, angiotensin-converting enzyme inhibitors, and mineralocorticoid receptor antagonists. He was noted to have atrial fibrillation (AF) and was on digoxin. His coronary risk factor management was good, however, he had exertional chest pain three-month prior to suffering OHCA. He underwent coronary CT angiography, which indicated calcification of the proximal left anterior descending (LAD) but no significant stenosis. He had not developed hospitalization for cardiovascular disease or suffered an episode of lethal arrhythmia or syncope.

**Table 1:** Summary of echocardiographic findings. LVDd, left ventricular end-diastolic diameter; LVDs, left ventricular end-systolic diameter; IVS, interventricular septum; PW, posterior left ventricular wall; LAD, left atrial dimension; EF, ejection fraction; DCT, deceleration time

Parameters	60 years	67 years	69 years (this admission)
LVDd (mm)	64	56	57
LVDs (mm)	50	45	46
IVS (mm)	12	11	9
PW (mm)	12	10	9
LAD (mm)	51	56	53
EF (%)	34	43.6	40
E/A	37/48	83/-	58/-
DCT (mm)	253	180	196
E/e’	7.7	11.1	8.1

**Figure 1:**
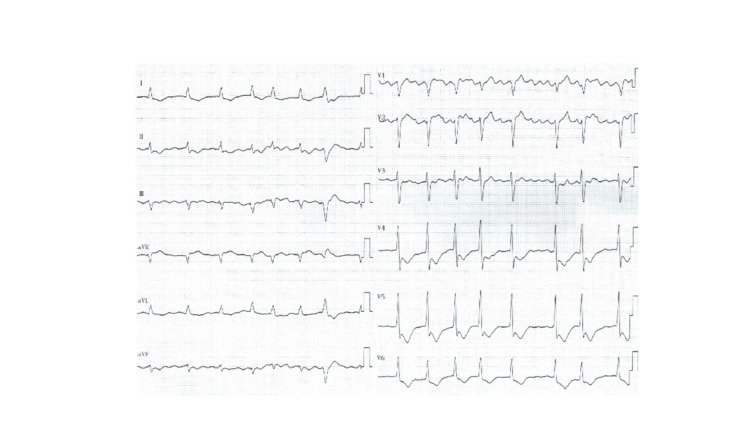
The ECG after ROSC showing atrial fibrillation rhythm, ST depressions in leads V4-6 and ST elevation in leads aVR and V1. ECG, electrocardiogram; ROSG, return of spontaneous circulation

 He was transferred to our emergency department under continuous cardiopulmonary resuscitation after experiencing OHCA at the train station. On arrival, an electrocardiogram (ECG) detected VF. He achieved a return of spontaneous circulation (ROSC) after three unsynchronized defibrillations (200J), and three epinephrine injections. Post ROSC, his blood pressure was 112/59 mmHg, heart rate 70 bpm, and oxygen saturation 99% under intubation on a fraction of inspiratory oxygen of 100%. Physical examination revealed coldness of the extremities, but not pulmonary rales and heart murmur. The ECG after ROSC showed the same AF rhythm as before, as well as new ST depression in leads V4-6 and ST elevation in leads aVR and V1 (Figure 1).

Laboratory findings showed no electrolyte abnormalities leading to potentially fatal arrhythmias (Table 2).

**Table 2:** Laboratory data on admission. WBC, white blood cell; RBC, red blood cell; CRP, C-reactive protein; TP, total protein; UA, uric acid; BUN, blood urea nitrogen; AST, aspartate aminotransferase; ALT, alanine aminotransferase; CK, creatine kinase; LD, lactate dehydrogenase; BNP, brain natriuretic peptide; NGSP, the National Glycohemoglobin Standardization Program; TG, triglyceride; HDL, high density lipoprotein; LDL, low density lipoprotein

Laboratory variables	Results	Reference values
WBC (/μL)	5400	4300-8000
Hemoglobin (g/dL)	12.6	12.4-17.2
CRP (mg/dL)	0.02	0-0.4
TP (g/dL)	5.7	6.6-8.1
Albumin (g/dL)	3.4	3.5-5.0
UA (mg/dL)	5.6	3.7-7.8
BUN (mg/dL)	19	8-20
Creatinine (mg/dL)	1.15	0.5-1.1
Sodium (mmol/L)	144	138-145
Potassium (mmol/L)	3.7	3.6-4.8
Chlorine (mmol/L)	101	101-108
Calcium (mg/dL)	9.2	8.8-10.1
Total bilirubin (mg/dL)	0.5	0.2-1.0
AST (U/L)	140	13-30
ALT (U/L)	138	8-42
CK (U/L)	97	59-248
CK-MB (U/L)	34	0-25
LD (U/L)	387	124-222
Troponin T (ng/dL)	0.028	0-0.014
BNP (pg/mL)	602.2	0-18.4
HbA1c (NGSP) (%)	5.7	4.6-6.2
TG (mg/dL)	31	50-150
HDL-cholesterol (mg/dL)	47	30-80
LDL-cholesterol (mg/dL)	36	70-139

**Figure 2:**
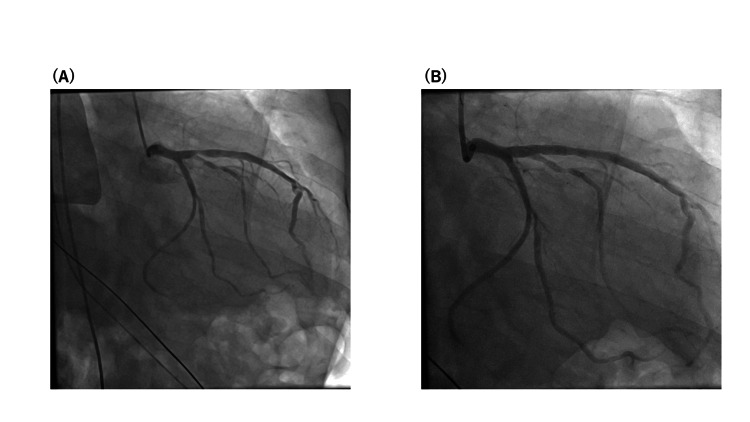
Coronary angiography. (A) Coronary angiography revealed severe coronary stenosis in the proximal LAD coronary artery. (B) PCI was successfully performed. PCI, percutaneous coronary intervention; LAD, left anterior descending

TTE showed diffusely reduced LV wall motion (EF=40%), but no regional wall motion abnormalities. He immediately underwent coronary angiography (CAG) to evaluate for the acute coronary syndrome (ACS). A 90% stenosis in the proximal LAD artery was diagnosed (Figure 2A), and optical frequency domain imaging showed fibrous plaques without rupture in culprit lesions. A percutaneous coronary intervention (PCI) was performed continuously (Figure 2B).

Creatine kinase (CK) and CK-MB peaked at 2452 and 63 U/L, respectively. It seemed that a high value of CK compared to CK-MB was due to muscle injury of cardioversion. He was extubated on day 7, and his neurological function fully recovered to his baseline.

**Figure 3:**
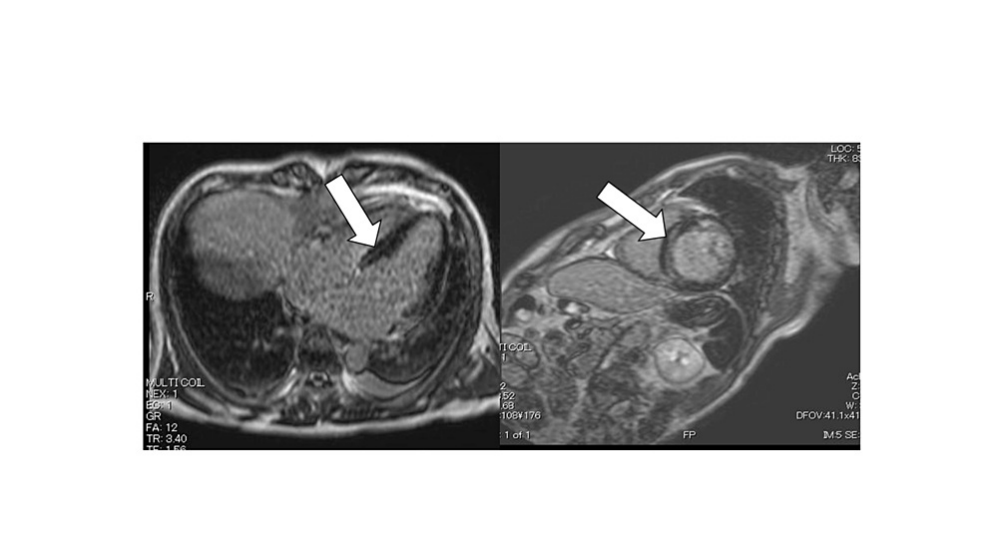
CMR images showing the presence of mid-wall LGE in IVS (white arrow). CMR, cardiac magnetic resonance; LGE, late gadolinium enhancement; IVS, interventricular septum

 Subsequently, the patient underwent a clinical examination to determine the etiology of potentially fatal arrhythmias. CMR imaging which took place about a week later revealed late gadolinium enhancement (LGE) in the interventricular septum (IVS) (Figure 3), and there were no other significant findings. This was a finding that was inconsistent with CAD in the LAD region. AF was another potential reason that can cause cardiomyopathy; however, we considered unlikely this possibility because LGE in the IVS is not a typical finding in AF. We could not be sure about the cause of VF and cardiopulmonary arrest (CPA) which is why serum levels of GH and serum IGF-1 were normal (2.38 and 142 ng/mL, respectively). However, the fact that there are CMR findings consistent with non-ischemic cardiomyopathy strongly suggests. Therefore, we implanted an ICD for secondary prevention of VF and CPA. After ICD implantation, the patient was discharged without developing potentially fatal arrhythmias. Heart failure or inappropriate ICD shocks and similar problems have not been noted since discharge.

## Discussion

Cardiovascular disease is known to be an important complication of acromegaly, accounting for increased morbidity and reduced life expectancy in these patients [2, 4]. This case was an example of VF due to acromegalic cardiomyopathy with CAD, which made it difficult to decide whether ICD should be implanted.

Implantable cardioverter-defibrillator is one of the most effective and established treatments to prevent sudden cardiac death due to potentially fatal tachyarrhythmia, and improve survival regardless of the type of heart disease. Japanese guidelines recommended that ICD may be considered for patients with non-ischemic cardiomyopathy even if VT/VF was "due to reversible acute causes (e.g., HF, electrolyte abnormality, or drugs), but whose possibility of re-exposure to the same conditions is high." In this case, we diagnosed fatal arrhythmia due to non-ischemic cardiomyopathy accompanied by ACS and, therefore, decided to implant an ICD as secondary prevention according to Japanese guidelines [5]. However, if ACS had been the only cause of OHCA, ICD would not have been indicated, because persistent VT and VF that appear in the acute phase of ACS are unlikely to recur after resolution of the myocardial ischemia and subsequent stabilization of the arrhythmic substrate.

The precise diagnosis in patients with CPA by VF is important because the indication for ICD must be determined by the underlying cardiac disease. In patients with acromegaly, approximately 3% have been reported to have a unique cardiomyopathy characterized by biventricular hypertrophy, myocardial necrosis, lymphocytic infiltration, and interstitial fibrosis [2]. The mechanisms of acromegalic cardiomyopathy have not been fully clarified, but long-term elevations in serum GH and IGF-I levels may cause myocardial hypertrophy and interstitial fibrosis of cardiomyocytes [2]. Furthermore, interstitial fibrosis may cause arrhythmia in acromegalic cardiomyopathy. Lombardi et al. and Suyama et al. reported that the incidence of premature ventricular contractions was significantly decreased by suppressing GH and IGF-1 with somatostatin analog treatment in patients with acromegaly [6-7]. However, Maffei et al. showed that the frequency of ventricular arrhythmia did not change after successful treatment of acromegaly [8]. This discrepancy may be due to a difference in the severity of myocardial interstitial fibrosis in both studies. Due to a notable correlation between GH and IGF-1 levels and interstitial fibrosis [9], achieving control of GH levels has been successful in improving prognosis [10]. Thus, not only the presence but also the degree of fibrosis is considered to be involved in the appearance of potentially fatal arrhythmias.

Moreover, in addition to optimal medical therapy for heart failure, the normalization of GH and IGF-1 levels, whether by surgical or pharmacological therapy, is essential to prevent or improve cardiovascular complications. Because the diagnosis of acromegaly is often delayed by many years due to the silent progression of the disease, the necessity of early recognition and management is paramount to avert chronic cardiovascular damage [11]. Patients with physical findings or past history suggestive of acromegaly should undergo echocardiography to avoid overlooking acromegalic cardiomyopathy. It has been recently reported that the IVS’s thickness, as seen by CMR, relates to both age and duration of illness in acromegalic patients; CMR is also useful for evaluating changes in cardiac structure and function in such patients [12]. Regarding CMR findings, CAD patients have subendocardial or transmural enhancement, while typical cardiomyopathy patients have longitudinal striae of mid-wall enhancement [13]. The CMR findings, in this case, show the latter. The findings of the former were absent, probably because of the trivial myocardial damage caused by CAD. Furthermore, by evaluating LGE after gadopentetic acid injection on CMR, fibrosis characteristically develops in the mid-myocardium of the left ventricle or IVS in patients with acromegaly [12]. Therefore, in our case, we could not be sure about the cause of VF, but the fact that there are CMR findings consistent with non-ischemic cardiomyopathy strongly suggests ICD implantation as secondary prevention. Thus, CMR is recommended as an accurate and comprehensive diagnostic method for patients with heart disease, including acromegalic cardiomyopathy.

## Conclusions

In conclusion, the prognosis of cardiac complications caused by acromegaly is poor, and ICD is one of the most effective and established treatments. Although it may be difficult to determine the indication for ICD in some cases, it is necessary to make an appropriate decision by making full use of diagnostic imaging such as CMR.
